# Plasma sterols and depressive symptom severity in a population-based cohort

**DOI:** 10.1371/journal.pone.0184382

**Published:** 2017-09-08

**Authors:** Basar Cenik, Can Cenik, Michael P. Snyder, E. Sherwood Brown

**Affiliations:** 1 Department of Psychiatry, The University of Texas Southwestern Medical Center, Dallas, Texas, United States of America; 2 Department of Genetics, Stanford University School of Medicine, Stanford, California, United States of America; McMaster University, CANADA

## Abstract

Convergent evidence strongly suggests major depressive disorder is heterogeneous in its etiology and clinical characteristics. Depression biomarkers hold potential for identifying etiological subtypes, improving diagnostic accuracy, predicting treatment response, and personalization of treatment. Human plasma contains numerous sterols that have not been systematically studied. Changes in cholesterol concentrations have been implicated in suicide and depression, suggesting plasma sterols may be depression biomarkers. Here, we investigated associations between plasma levels of 34 sterols (measured by mass spectrometry) and scores on the Quick Inventory of Depressive Symptomatology-Self Report (QIDS-SR_16_) scale in 3117 adult participants in the Dallas Heart Study, an ethnically diverse, population-based cohort. We built a random forest model using feature selection from a pool of 43 variables including demographics, general health indicators, and sterol concentrations. This model comprised 19 variables, 13 of which were sterol concentrations, and explained 15.5% of the variation in depressive symptoms. Desmosterol concentrations below the fifth percentile (1.9 ng/mL, OR 1.9, 95% CI 1.2–2.9) were significantly associated with depressive symptoms of at least moderate severity (QIDS-SR_16_ score ≥10.5). This is the first study reporting a novel association between plasma concentrations cholesterol precursors and depressive symptom severity.

## Introduction

Major depressive disorder (MDD) is a highly prevalent disease and a leading cause of disability worldwide [1]. MDD is heterogeneous in its etiology and clinical characteristics [2]. Depression biomarkers hold potential for identifying etiological subtypes, improving diagnostic accuracy, predicting treatment response, and personalization of treatment. Changes in various biochemical pathways have been associated with MDD, including inflammatory, neurotrophic, and hypothalamic-pituitary-adrenal (HPA) axis alterations [3]. In contrast, plasma lipids have received relatively little attention as depression biomarkers despite a long-recognized association between low plasma cholesterol, suicidal behavior, and depression [4–11]. Human plasma contains a large number of lipids that have not been systematically investigated [12]. Twenty-nine of these lipids, as well as commonly assessed lipoprotein fractions, were recently quantified with mass spectrometry in a large, ethnically diverse, phenotypically well-characterized, population-based sample in the Dallas Heart Study (DHS) [13]. Between 2001 and 2012, more than 115 reports have been published based on the DHS data, mostly on cardiovascular outcomes and genetics of intermediary metabolism. Most relevant to our study, Stiles et al. [13] screened more than 60 sterol species and identified 29 that are consistently detected in human plasma. Of these 29, plasma concentrations of several sterols varied inter-individually, and by age, gender, and ethnicity. Some of these sterols, namely sitosterol, campesterol, stigmasterol, and stigmastanol, are plant sterols thought to be dietary in origin. Stiles et al. also identified 16 genetic loci correlated with concentrations of 19 sterols in a genome wide association study in this sample. Remarkably, many of these sterols are under-studied despite the central role played by cholesterol and its metabolites in human metabolism and disease.

There is ample reason to hypothesize that sterols may be associated with depression. Low plasma and postmortem brain cholesterol concentrations have been found to correlate with increased risk for depression and suicidal behavior. [4, 5, 8, 10, 11]. Oxysterols, oxygenated derivatives of cholesterol, modulate the Sonic Hedgehog pathway, NMDA receptors, and the expression of nuclear receptors in retinal neurons, in addition to being intermediates in cholesterol catabolism, potential regulators of cholesterol homeostasis, and transport forms of cholesterol. Some oxysterols accumulate in postmortem brains from Alzheimer disease patients [14–16]. Vitamin D, a secosteroid with potential roles in brain development, adult brain function, and neuropsychiatric illness has also been linked to depression [17].

In this study, we hypothesized that concentrations of plasma lipids, including oxysterols, sterol and secosteroid metabolism intermediaries, lipoproteins and dietary sterols are predictive of depressive symptom severity scores in the DHS sample. This is the first DHS report examining plasma sterols in relation to depression.

## Materials and methods

### Study population

This investigation was conducted as part of the Dallas Heart Study (DHS). DHS was undertaken in 2000 as a single-site (Dallas County, Texas, USA), multiethnic, cross-sectional, population-based study of cardiovascular health. The Dallas Heart Study 2 (DHS-2) is a longitudinal follow-up study of a subset of participants who returned from 2007 onwards for a second clinical examination, an extensive health survey, laboratory testing and imaging studies. Of note, DHS was a probability-based, epidemiological sample representative of Dallas County, except for intentional over-sampling of African American ethnicity (~52% of subjects who provided a plasma sample); the subjects were not selected based on presence of heart disease or any cardiovascular risk factors. Sampling design, recruitment procedures and other details of the study population have been published [13, 18]. Out of the 3402 subjects included in DHS-2 sample, plasma sterol concentrations were available for 3228. Of those, we included data from the 3117 participants who had completed at least one item on the QIDS-SR_16_ scale.

This study was approved by the Institutional Review Board of The University of Texas Southwestern Medical Center as part of the Dallas Heart Study and all subjects provided written informed consent after receiving a complete description of the study.

The Executive Committee of the Dallas Heart Study has imposed restrictions on sharing the de-identified data set due to the potential loss of anonymity of participants. Requests for access to the data can be addressed to Dr. Helen H. Hobbs, Director, Donald W. Reynolds Cardiovascular Clinical Research Center, The University of Texas Southwestern Medical Center, 5323 Harry Hines Boulevard NB10.204A MS 8591, Dallas, Texas 75930.

### Measurement of sterol and lipoprotein concentrations

Data on sterol and lipoprotein concentrations was obtained from the DHS executive committee. The mean, median, and range for all sterol concentrations have been previously published and can be found in Table 1 of Stiles et al. [13].

**Table 1:** 34 plasma sterols and lipoproteins investigated in this study.

14-Desmethyl lanosterol
22R-Hydroxycholesterol
24,25-Epoxycholesterol
24-Dihydrolanosterol
24-Oxocholesterol
24S-Hydroxycholesterol
25-Hydroxycholesterol
25-Hydroxyvitamin D_2_
25-Hydroxyvitamin D_3_
27-Hydroxycholesterol
4β-Hydroxycholesterol
5,6α-Epoxycholesterol
5,6β-Epoxycholesterol
5α-Hydroxycholesterol
7-Dehydrocholesterol
7-Oxocholesterol
7α,27-Dihydroxycholesterol
7α-Hydroxycholesterol
8-Dehydrocholesterol
Campesterol
Cholestanol
Cholestenone
Desmosterol
HDL Cholesterol
Lanosterol
Lathosterol
LDL Cholesterol
Sitosterol
Stigmastanol
Stigmasterol
Total Cholesterol
Triglycerides
VLDL Cholesterol
Zymosterol

Briefly, blood samples were collected in the fasting state; deuterated standards were added to plasma; lipids were extracted from saponified plasma; the sample was hydrolyzed, followed by solid phase extraction; compounds were resolved with high performance liquid chromatography and gas chromatography (GC) and quantified by quadrupole mass spectrometry (MS) or GC-MS. The complete methods and assay characteristics have been published [13, 19, 20]. The 34 plasma sterols and lipoproteins shown in Table 1 were included in this study.

### Measurement of depressive symptom severity

Depressive symptom severity was measured using QIDS-SR_16_, a validated, widely-used, self-report instrument [21–25]. The scale is freely available online at http://www.ids-qids.org/tr-english.html. This scale is composed of 16 questions, rated 0 to 3. For questions 1 to 4, 6 to 9, and 15 to 16, only the highest rating is taken into account for the final score, resulting in a scale composed of 9 items corresponding to the 9 criteria for major depressive episodes, scored 0 to 3, for a maximum possible score of 27. The mean scores and standard deviations for all 9 items are shown in Table 2.

**Table 2:** Mean scores and standard deviations for 9 items comprising the QIDS-SR_16_.

Item (Symptom)	Mean	Standard Deviation
1–4 (Sleep)	2.034	0.865
5 (Sadness)	0.523	0.743
6–9 (Appetite)	1.051	1.099
10 (Concentration)	0.402	0.649
11 (View of self)	0.243	0.688
12 (Suicidality)	0.082	0.356
13 (Interest)	0.258	0.609
14 (Energy)	0.494	0.729
15–16 (Psychomotor)	0.534	0.887

86.3% of subjects had completed all items on QIDS-SR_16_, while 9.5% had left exactly one item blank. The remaining 4.1% had two or more missing items. To address the missing values, we employed a k-nearest neighbor method for imputation [26]. Eleven subjects had answered less than half the items. For these eleven subjects, we assigned the mean score for that item instead of the missing value. After the imputation step, we calculated the total QIDS-SR_16_ scores as previously described [21]. Although scores on QIDS-SR_16_ are normally integers, the imputation method assigns decimals; therefore, we considered scores ≥10.5 as indicative of depressive symptoms of at least moderate severity. Depressive symptom severity was modeled continuously for primary analysis and dichotomized only for secondary analysis.

### Statistical analysis and random forest classifier training

All statistical analyses were carried out in R software (version 3.2.5 [2016-04-14]). As predictor variables for QIDS-SR_16_ score, we used 34 sterol and lipoprotein concentrations and nine demographic and general health related parameters. All sterol concentrations, as well as age, formal education in years, and number of chronic illnesses were treated as quantitative variables. Number of chronic illnesses was based on self-report and defined as the number of positive responses to the question “Has a doctor ever told you that you have X?” where X is one of the following: “heart attack”, “stroke”, “heart failure”, “diabetes”, “hypertension”, “emphysema”, “hepatitis”, “kidney disease”, “lupus”, “rheumatoid arthritis”, “inflammatory bowel disease”, “pulmonary sclerosis”, “Alzheimer disease”, “obstructive sleep apnea”, or “cancer”. Yearly household income was categorized into 10 income brackets, 1 denoting the lowest income and 10 denoting the highest income, and used as a quantitative variable. Gender, ethnicity (Hispanic, non-Hispanic white, non-Hispanic black, or other) and marital status (married, living as married/living with partner, separated/divorced, widowed, or never married) were treated as categorical variables. Drinking status was classified into four categories, “lifelong abstainer”, “recent abstainer”, “low risk drinking”, or “high risk drinking”. High risk drinking was defined as more than 14 standard drinks per week for men and more than 7 standard drinks per week for women, based on Centers for Disease Control and Prevention recommendations. Smoking status was analyzed categorically: “never smoker”, “past smoker”, or “current smoker”.

The sterol levels are correlated with each other [13]. Furthermore, given the biochemical synthesis pathways, the levels of certain sterols are conditionally dependent on each other. Therefore, a simple regression framework is unsuitable and we instead adopted a random forest approach for modeling [27]. Random forest models were constructed using “randomForest” library in R [28]. We assessed the prediction performance using explained variation as defined by

$$1-{\sum \left(y-\hat{y}\right)}^{2}/\sum {\left(y-\mu_{y}\right)}^{2}$$

where y is the QIDS-SR_16_ score, μ_y_ is the mean QIDS-SR_16_ score, and ŷ is the predicted score from the random forest model.

To assess feature importance, we used a permutation based approach. Specifically, values of each variable is permuted while holding the other variables constant and the percent increase in mean squared error for out-of-bag samples is calculated. Note that by definition, this approach can underestimate the importance of correlated variables [27].

For feature selection, we used the ‘rfe’ function in the “caret” R package [29]. Specifically, we employed a recursive feature elimination approach using a 5-fold repeated cross-validation scheme. We used the variation explained as the metric to be maximized. We allowed for either 1% or 5% tolerance to ensure a balance between performance and the number of selected variables. We utilized the “doMC” R package to parallelize the feature selection [30]. In this validation approach, the scoring for a subject as a function of sterol measurements was performed using a model that had not been trained on that subject, enabling the separation of training and validation samples in an unbiased way.

**Fig 1:**
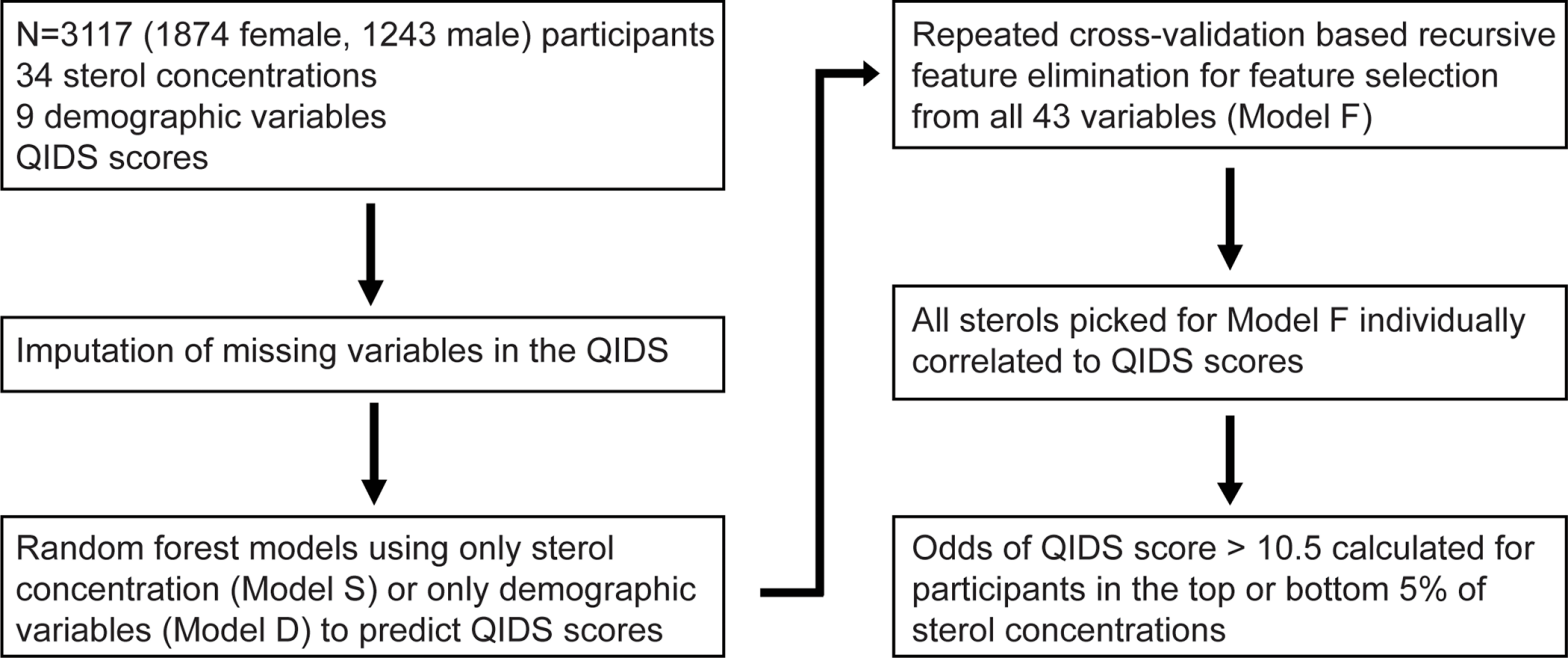
Pre-specified data analysis flowchart. The models S, D, and F are further specified in Table 4. QIDS, Quick Inventory of Depressive Symptomatology-Self Report.

In secondary analysis, we defined depressive symptoms of at least moderate severity as corresponding to QIDS-SR_16_ scores ≥10.5. We used Fisher’s exact test to calculate the odds ratios and *p*-values of association between selected sterol concentrations and depressive symptoms of at least moderate severity. Spearman’s method was used to calculate correlation coefficients. *p* values were corrected for multiple comparisons with Holm’s method. Fig 1 summarizes the pre-specified data analysis plan.

## Results

We analyzed data from 3117 adults (1874 females, 1243 males) from the Dallas Heart Study (DHS)-2 cohort. First, we determined that 13.7% of the participants had not answered one or more questions on the QIDS-SR_16_. To assess any potential systematic differences between subjects that completed all questions and those that left at least one question blank, we carried out two complementary analyses. First, for each question, we compared the score distribution of subjects with no missing values and at least one missing value. We found that, except for item 14 (Energy level: 0: There is no change in my usual level of energy. 1: I get tired more easily than usual. 2: I have to make a big effort to start or finish my usual daily activities. 3: I really cannot carry out most of my usual daily activities because I just don’t have the energy.), there was no statistically significant difference between the two groups (Bonferroni-corrected chi-squared test, *p* >0.05). Second, we analyzed the mean score on questions that were completed. For individuals that completed the entire questionnaire, this is simply their total score divided by nine. Subjects who left more than three questions blank had higher per question severity scores (median score 0.56 for subjects with no missing items versus 1.5 for subjects with more than 3 missing items; Wilcoxon rank sum test, *p* = 1x10^-10^). These systematic differences are consistent with more depressed subjects being less likely to complete the entire questionnaire. Hence, we decided to impute the missing values since excluding these potentially more severely depressed subjects would have biased our results and reduced the power of our analysis. Nonetheless, we repeated all analyses presented in the manuscript using only subjects that completed the entire questionnaire (i.e., without imputation) and observed qualitatively consistent results for all reported findings (data not shown).

Median score on the QIDS-SR_16_ was 5 (range 0–24, S1 Fig) and 11.6% of subjects had scores greater than 10.5 (corresponding to depressive symptoms of at least moderate severity) after imputation of missing values. This figure is roughly consistent with the reported 12-month prevalence of major depression (5–10%) in North America [31, 32]. Other characteristics of the subjects are summarized in Table 3.

**Table 3:** Characteristics of study population.

	Number of subjects	3117
Gender	Female (%^1^)	60
Age	Median (range)	50 (18–85)
Ethnicity (%^1^)	Black	51
	Non-Hispanic White	33
	Hispanic	14
	Other	2
Annual household income	25^th^ percentile	$20,000-$25,000
	Median	$35,000-$40,000
	75^th^ percentile	$50,000-$75,000
Formal education in years	Median (range)	13 (0–16)
High-risk alcohol use	Percentage^1^	7
Current smoker	Percentage^1^	22
Number of chronic illnesses	Median (range)	1 (0–7)
QIDS-SR_16_ score^2^	Median (range)	5 (0–24)
	≥ 10.5 (%)	11.6

^1^, rounded to the nearest integer

^2^, after imputation of missing items

Next, we investigated whether plasma sterol concentrations could be used to predict depressive symptom severity as assessed by the QIDS-SR_16_. We treated QIDS-SR_16_ scores as a quantitative variable. Plasma sterol concentrations are known to be highly correlated with each other [13]. Given the dependencies between the sterol concentrations, we decided to avoid standard regression-based approaches and used a machine learning approach to model the relationship between depression symptom severity and plasma sterol concentrations. We constructed a random forest model (RFM) using concentrations of 34 different sterols and lipoproteins. This model explained 4.2% of the variation in QIDS-SR_16_ scores in the study population (Model S in Table 4). Table 5 shows all 34 variables used in the model ranked by importance according to percent increase in mean squared error metric.

**Table 4:** Summary of random forest models.

Model	Variables used	Number of Variables	% Variation in QIDS-SR_16_ Scores explained
S	Sterol concentrations	34	4.2
A	Age, gender, and ethnicity	3	2.2
S+A	Sterol concentrations, age, gender, and ethnicity	37	7.4
D	Demographics and general health indicators	9	12.8
F	Selected features from models S and D	19	15.5

**Table 5:** Variables used in random forest model *s*, ranked in order of importance.

Sterol	% Increase in mean squared error
Desmosterol	37.19
14-Desmethyl lanosterol	27.35
Sitosterol	27.24
Stigmasterol	25.41
Lathosterol	25.03
Lanosterol	24.79
7-Dehydrocholesterol	23.93
Cholestanol	22.36
7-Oxocholesterol	22.11
25-Hydroxycholesterol	20.97
Campesterol	20.96
25-Hydroxyvitamin D_3_	19.94
Cholestenone	19.63
5α-Hydroxycholesterol	18.87
5,6β-Epoxycholesterol	18.43
Total Cholesterol	17.91
4β-Hydroxycholesterol	16.55
24-Dihydrolanosterol	16.23
22R-Hydroxycholesterol	15.67
Zymosterol	15.16
24,25-Epoxycholesterol	14.40
5,6α-Epoxycholesterol	13.39
LDL Cholesterol	12.99
7α-Hydroxycholesterol	12.91
8-Dehydrocholesterol	12.73
HDL Cholesterol	11.78
24S-Hydroxycholesterol	10.74
24-Oxocholesterol	9.85
27-Hydroxycholesterol	9.49
VLDL Cholesterol	9.41
Triglycerides	8.68
7α,27-Dihydroxycholesterol	6.97
Stigmastanol	5.88
25-Hydroxyvitamin D_2_	0.96

Plasma concentrations of several sterols have been reported to correlate with gender and ethnicity [13], both of which are associated with depression prevalence [31]. Hence, it is possible that the predictive power of sterols is solely driven by their relationship to gender and ethnicity. To test this hypothesis, we first built a model using only age, gender, and ethnicity as variables (Model A in Table 4). These three variables explained only 2.2% of the variation in QIDS-SR_16_ scores. To find out whether sterol concentrations provide any further predictive information on top of age, gender, and ethnicity, we combined these three demographic variables with the sterol concentrations to build an RFM with 37 variables (Model S+A in Table 4); this model explained 7.4% of the variation in QIDS-SR_16_ scores, an apparently additive improvement in predictive accuracy. The variable with the highest importance by percent increase in mean squared error metric in this model was gender, followed by desmosterol concentration.

**Fig 2:**
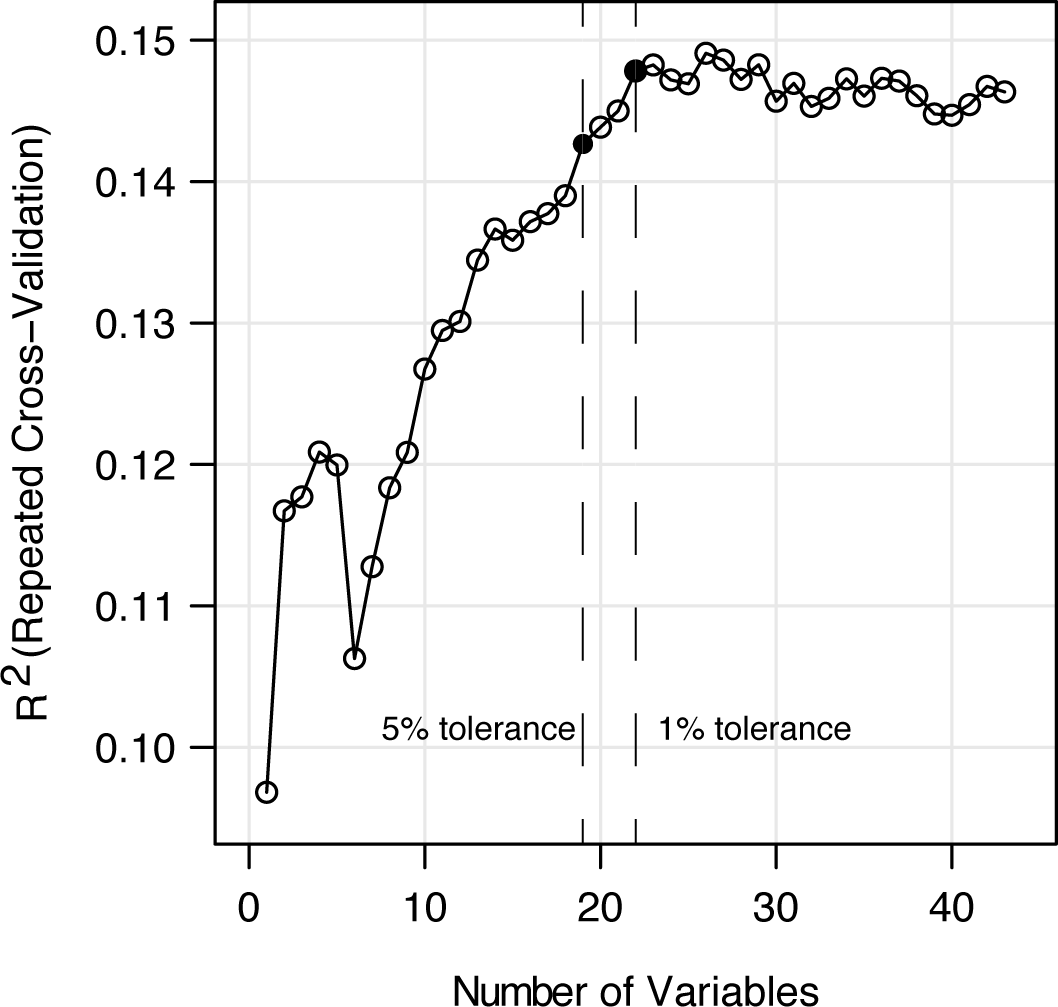
Unbiased feature selection using a recursive elimination method. A total of 34 sterol concentration measurements and 9 demographic parameters were used to predict QIDS-SR16 scores from 3117 subjects. A repeated cross-validation approach is used such that data is partitioned into test and training sets via resampling. Performance as measured by percent variation explained as a function of the number of features used in training the RFM is shown.

**Fig 3:**
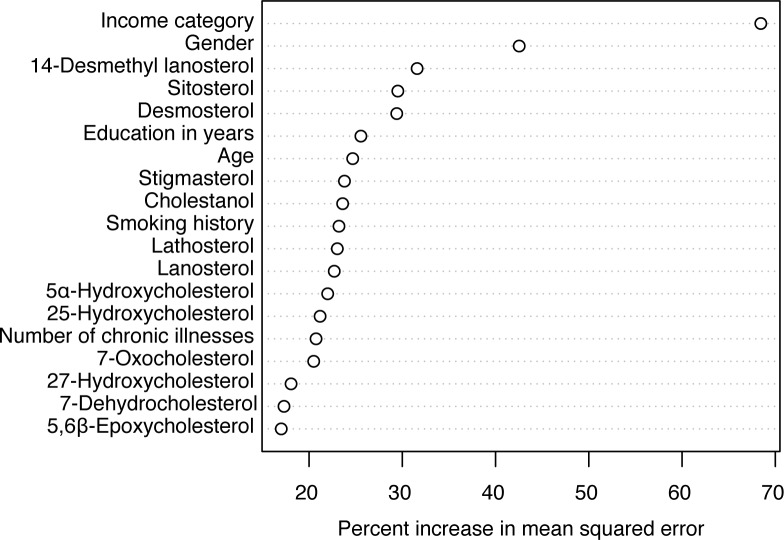
Nineteen variables comprising Model F. Parameters selected from a pool of 43 demographic or general health variables and sterol concentrations by a feature selection algorithm based on explained variation in QIDS-SR16 scores is shown, ranked by importance judged by the percent increase in mean squared error metric.

For plasma biomarkers to be clinically useful, they should have predictive value over and above known risk factors for depression. To test this, we constructed Model D from nine demographic and general health variables previously suggested to be correlated with depression risk and available in the DHS-2 dataset: gender, ethnicity, age, household income, education in years, alcohol use, smoking status, number of chronic illnesses, and marital status [32, 33]. The RFM constructed from these nine variables explained 12.8% of the variation in QIDS-SR_16_ scores. Next, we wanted to test whether inclusion of a subset of sterols may further improve the predictive power of a model that uses the known demographic risk factors. Hence, we employed a repeated cross-validation based recursive feature elimination scheme to determine a subset of these 43 variables (34 sterols + 9 demographics). We adopted a random forest validation approach, such that the scoring for subject as a function of sterol measurements was performed using a model that had not been trained on that subject. Hence, our method enables the separation of training and validation samples in an unbiased way. We set a tolerance threshold of 5% increase in variation explained to balance parsimony with explained variation. This model yielded 19 variables, 13 of which were sterol concentrations. For comparison, setting the threshold at 1% would have yielded 22 variables (Fig 2). An RFM constructed with these 19 variables explained 15.5% of the variation in QIDS-SR_16_ scores, better than all the other models tested (Model F, Table 4). Household income was the most important variable predicting depressive symptom severity scores, followed by gender and the concentrations of 14-desmethyl lanosterol, sitosterol, and desmosterol (Fig 3). Next, we applied Model F to female-only and male-only subsets of the dataset. Model F explained 13.7% of the variation in QIDS scores in the female-only sample and 6.9% of the variation in the male only sample. Few male participants had QIDS scores >10.5, likely limiting the power of the analysis. The most important variables in the female-only model were income category, smoking status, age, education, 14-desmethyl-lanosterol, 7-dehydrocholesterol (7-DHC), sitosterol, lathosterol, 5-6-β-hydroxycholesterol and desmosterol, which is qualitatively similar to the results from the full dataset.

**Fig 4:**
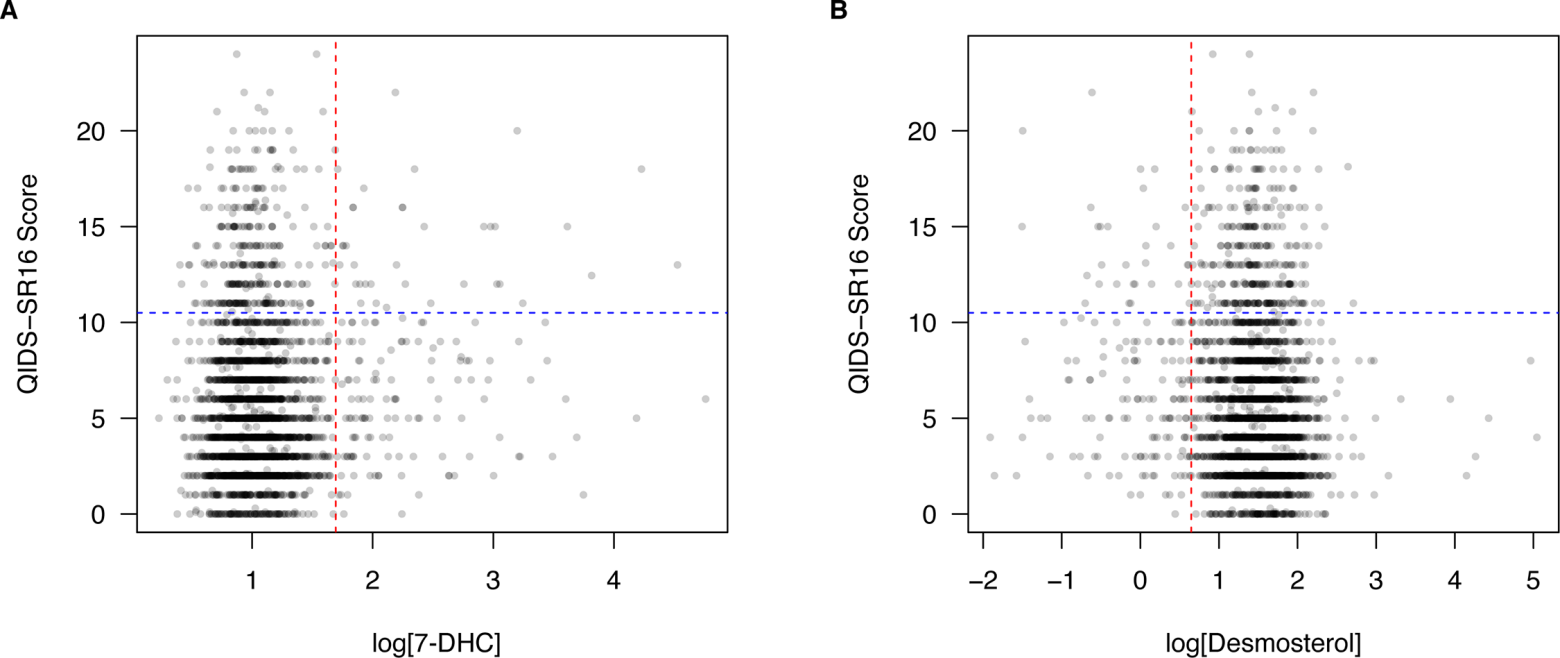
Correlation of QIDS-SR_16_ scores with 7-DHC and desmosterol concentrations. (a), 7-DHC; (b), desmosterol. Blue dashed line indicates QIDS-SR_16_ score of 10.5, red dashed line indicates 95^th^ percentile for 7-DHC and 5^th^ percentile for desmosterol concentrations. Each gray circle indicates one data point.

Finally, we wanted to explore the interpretability of our model. For this purpose, we tested whether any of the 13 sterol concentrations picked by the feature selection algorithm were individually correlated with QIDS-SR_16_ scores. Concentrations of desmosterol, 5,6β-epoxycholesterol, and 25-hydroxycholesterol were significantly correlated with QIDS-SR_16_ scores after Holm’s correction for multiple comparisons, while adjusted *p* values for cholestanol and 14-desmethyl lanosterol were close to significance (Table 6). For each of these 13 sterols, we also calculated odds ratios for having a QIDS-SR_16_ score greater than 10.5 by setting a threshold at either the 5^th^, or the 95^th^ percentile of sterol concentrations based on the sign of the correlation coefficient. Concentrations of 7-DHC above the 95^th^ percentile (5.4 ng/mL, OR 2.4, 95% CI 1.6–3.6, adjusted-*p* <0.001) and desmosterol concentrations below the 5^th^ percentile (1.9 ng/mL, OR 1.9, 95% CI 1.2–2.9, adjusted-*p* 0.053) were associated with depressive symptoms of at least moderate severity, albeit the multiple hypothesis testing corrected *p* value for desmosterol was on the margin of significance. An association between higher cholestanol concentrations and depressive symptom severity was not statistically significant following correction (Table 6). Fig 4 depicts the correlations between QIDS-SR_16_ scores and 7-DHC (panel a) and desmosterol (panel b) concentrations.

**Table 6:** Odds ratios and correlation coefficients for six of the 13 sterols picked by feature selection.

	Odds of QIDS-SR_16_ score ≥ 10.5				Correlation with QIDS-SR_16_ Score	
Sterol	Cut-off	OR	95% CI^1^	*p-adjusted*^1^	Spearman’s rho	*p-adjusted*^1^
7-Dehydrocholesterol	> 5.4 ng/mL^2^	2.4	1.6–3.6	<0.001	0.03	0.639
Desmosterol	< 1.9 ng/mL^3^	1.9	1.2–2.9	0.053	-0.11	<0.001
Cholestanol	> 23.5 ng/mL^2^	1.6	1.0–2.5	0.316	0.05	0.058
5,6β-Epoxycholesterol	> 2.7 ng/mL^2^	1	0.6–1.7	1	0.05	0.028
25-Hydroxycholesterol	> 0.1 ng/mL^2^	1.1	0.7–1.8	1	0.06	0.006
14-Desmethyl lanosterol	< 1.2 ng/mL^3^	1.3	0.8–2.1	1	-0.05	0.064

^1^, *p* values are corrected for 13 comparisons using Holm’s method. Confidence intervals are based on Fisher’s exact test.

^2^, 95^th^ percentile

^3^, 5^th^ percentile

OR, odds ratio; CI, confidence interval

## Discussion

The contribution of derangements in sterol metabolism to depression is unknown. In this study, we used a machine learning approach to investigate whether plasma sterols concentrations are associated with depressive symptoms in an epidemiological cohort. We found novel associations between several sterols and depressive symptom severity.

**Fig 5:**
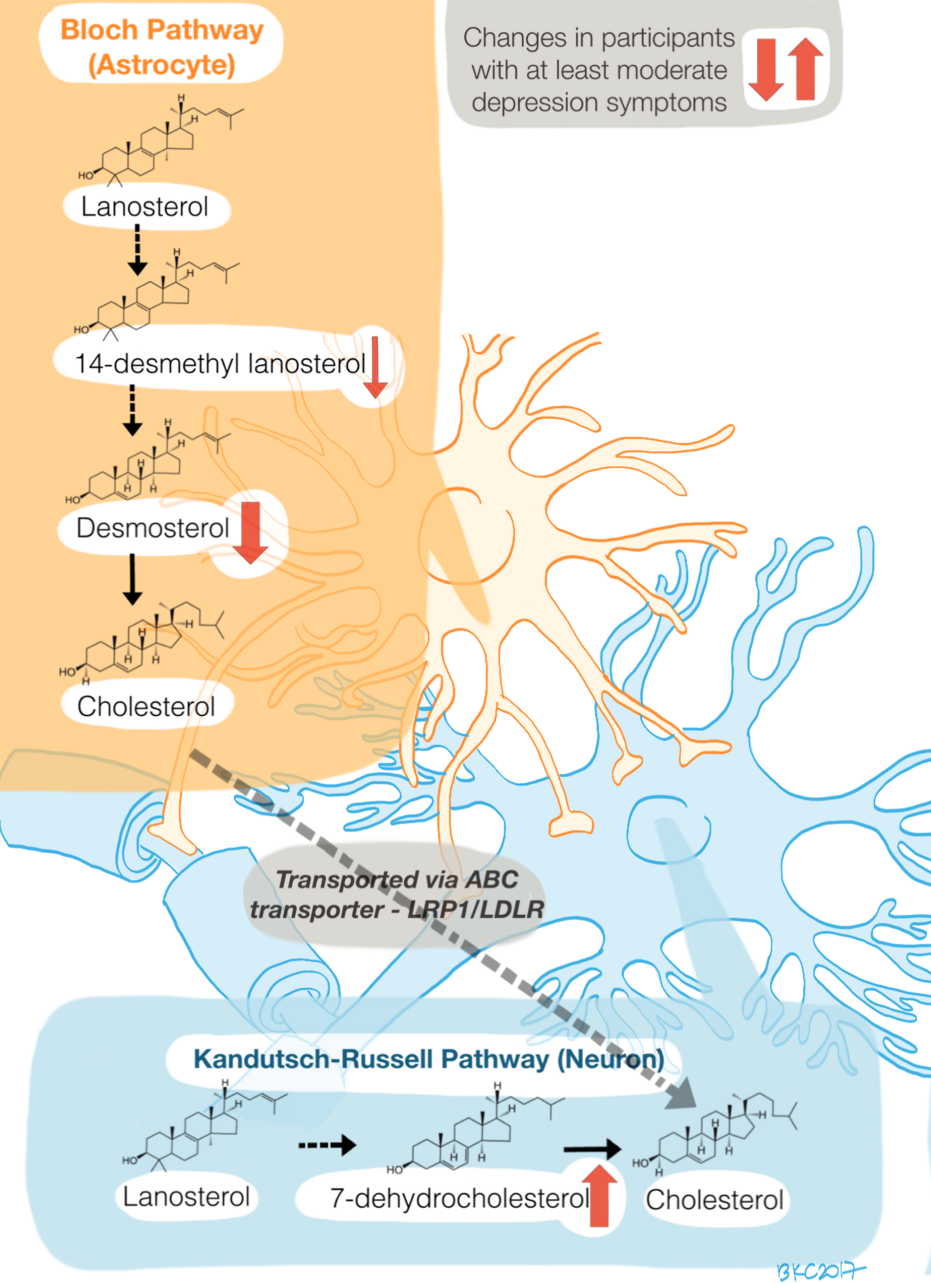
Hypothetical model of sterol derangements in depression. Arrows summarize main findings of the study (Table 6): Desmosterol concentrations are negatively correlated with depressive symptoms and low (<5^th^ percentile) desmosterol concentrations predict moderate to severe depressive symptoms. 7-DHC concentrations above the 95^th^ percentile also predict moderate to severe depressive symptoms. 14-Desmethyl lanosterol concentrations trend towards a negative correlation with depressive symptoms (not statistically significant after correction of *p* values for multiple comparisons).

Desmosterol and 7-DHC are the immediate precursors in the two main cholesterol synthesis pathways in mammals: 7-DHC in the Kandutsch-Russell pathway and desmosterol in the Bloch pathway (Fig 5). In the adult mammalian brain, the Kandutsch-Russell pathway is predominant in neurons, while the Bloch pathway is predominant in astrocytes. Cholesterol is thought to be synthesized largely in astrocytes and transported to the neurons via apolipoprotein E [34]. We found that increased 7-DHC (precursor in the neuronal pathway) and decreased desmosterol (precursor in the glial pathway) are associated with higher depressive symptom severity scores, suggesting an imbalance of cholesterol synthesis via the Kandutsch-Russell versus Bloch pathways. Additionally, an earlier precursor in the Bloch pathway, 14-desmethyl lanosterol, also trended towards a negative correlation with depression scores, similar to desmosterol concentrations, further strengthening the hypothesis that decreased concentrations of the Bloch pathway intermediaries correlate with higher depressive scores. Accumulation of 7-DHC is the hallmark of Smith-Lemli-Opitz Syndrome (SLOS), an autosomal recessive disorder of cholesterol synthesis that results from mutations in the gene coding for the enzyme, 7-DHC reductase, and manifests with aggressive and self-injurious behaviors [35].

Vitamin D is synthesized from 7-DHC in the human skin in a reaction that requires UV light [36]. Vitamin D deficiency has been associated with depression [17]. We included 25-Hydroxyvitamins D_2_ and D_3_ in the modelling. While 25-Hydroxyvitamin D_3_ was one of the predictive variables in Model S, it was not picked by feature selection when we included other demographic and health related variables.

Low cholesterol levels have long been associated with suicidal behavior [5, 6, 10]. In one study, low post-mortem brain cholesterol was associated with violent suicides [11]. An association between low plasma [7, 9] or brain [4] cholesterol and depression has also been reported. The underlying mechanism remains unknown. One hypothesis is that the link between brain cholesterol and impulsive-suicidal behavior is modulation of neurotransmitter signaling by cholesterol in lipid rafts, membrane microdomains rich in cholesterol [37, 38]: Key neurotransmitter receptors including 5-HT_1A_, 5-HT_2_, and D_1_ localize to lipid rafts and cholesterol content of neural membranes and localization of receptors to lipid rafts may modulate the function of these receptors [39]. Singh et al., [39] have suggested that either cholesterol or desmosterol is required for optimal ligand binding to the 5-HT_1A_ receptor. Similarly, lipid rafts may be necessary for the brain derived neurotropic factor (BDNF) signaling on nerve growth cones [40], intriguing given BDNF’s well-known association with depression [41]. Another potential link between cholesterol metabolism and brain function is neurosterols and oxysterols, both of which are synthesized from cholesterol [42]. Oxysterols, as well as 7-DHC, modulate hedgehog signaling, a signaling pathway involved in brain development and maintenance of hippocampal neurogenesis and may be modulated by antidepressants and antipsychotics [43]. It remains to be elucidated whether modulation of this pathway by cholesterol content of brain membranes plays a role in depression.

The changes in sterol concentrations that we correlated with depressive symptoms may be either state or trait markers. While it is not possible to rule out either based on a cross-sectional study, the latter might be more likely. Plasma sterol measurements are unlikely to directly reflect brain cholesterol metabolism, given that cholesterol does not ordinarily pass the blood-brain barrier [44]. The 7-DHC/desmosterol bio-signature probably hints at a metabolic disposition, perhaps genetically determined, that predisposes to depression. Indeed, in their analysis of this same DHS cohort, Stiles et al. identified genetic loci significantly linked to desmosterol and 7-DHC levels [13]. Future studies may explore the potential connection between these genetic loci and depression.

This study has several limitations. Firstly, this was an unbiased, hypothesis-generating analysis of epidemiological level data. We did not intend to develop a clinically useful biomarker panel at this stage and the population we studied was not a clinical sample. Rather, we have discovered previously unknown associations between several sterols and depression that may, in the future, contribute to development of such a panel, but also is hinting at novel biology on its own. Given the cross-sectional nature of our study, the mechanistic and diagnostic/clinical impact of our findings will have to be clarified by future clinical and experimental studies. Furthermore, correlational results from a cross-sectional study obviously do not prove causality, hence the changes we observed in sterol concentrations could be a result of depression, changes in diet, or medications.

Several psychotropic medications have been shown to alter 7-DHC levels and metabolism. Most relevant to our findings, Korade et al., recently reported that the use of aripiprazole, haloperidol and trazodone in psychiatric patients and controls is correlated with increased 7-DHC levels in plasma [45]. Aripiprazole is a second generation antipsychotic that is sometimes used as an augmentation agent in depression resistant to first line therapies. Trazodone, a selective serotonin reuptake inhibitor at high doses, is rarely used as an antidepressant currently, but is commonly prescribed as a sleep aid. Therefore, the elevated 7-DHC concentrations we observed in depressed participants could be explained by this confounder. On the other hand, Lauth et al., had reported that imipramine, clozapine, haloperidol and chlorpromazine induce the expression of 7-DHC reductase, the enzyme that converts 7-DHC to cholesterol, leading to modulation of hedgehog signaling [43]. Another study reported sterol regulatory element-binding protein induction by antidepressants [46]. It remains to be determined whether the effects of psychotropic medications on lipid metabolism are pleiotropic or related to their therapeutic effects. It would be interesting to test whether sterol concentrations predict response to different antidepressants in future studies. Finally, peripheral measurements of biomarkers, while advantageous in terms of feasibility and acceptability, are nevertheless one step removed from concentrations in the brain.

In summary, our study underscores the promise of unbiased machine learning approaches to biomarker discovery in depression. We identified novel leads for biomarker development in depression, under-studied sterols that are independently associated with depressive symptoms and, when deranged, confer risk for depressive symptoms of at least moderate severity in a large epidemiological cohort. Our findings suggest that an imbalance between glial versus neuronal cholesterol synthesis may be implicated in depression. Further studies may expand the role of lipid biosynthetic pathways in psychiatric illness from the known association of rare genetic defects causing severe behavioral disturbances to potential determinants of population-level risk for moderate depression. Future prospective studies of these sterols as depression biomarkers, as well as basic science investigations into their biology and genetic determinants seem warranted. Particularly, it would be very informative to conduct prospective studies investigating the change in plasma sterol concentrations versus depressive scores over time.

## Supporting information

S1 Fig Distribution of QIDS scores. (TIF)
