# Supplementary figures and images for: Plasma sterols and depressive symptom severity in a population-based cohort
Source: PLoS One. 2017 Sep 8;12(9):e0184382. doi: 10.1371/journal.pone.0184382 (PMC5590924; doi:10.1371/journal.pone.0184382)

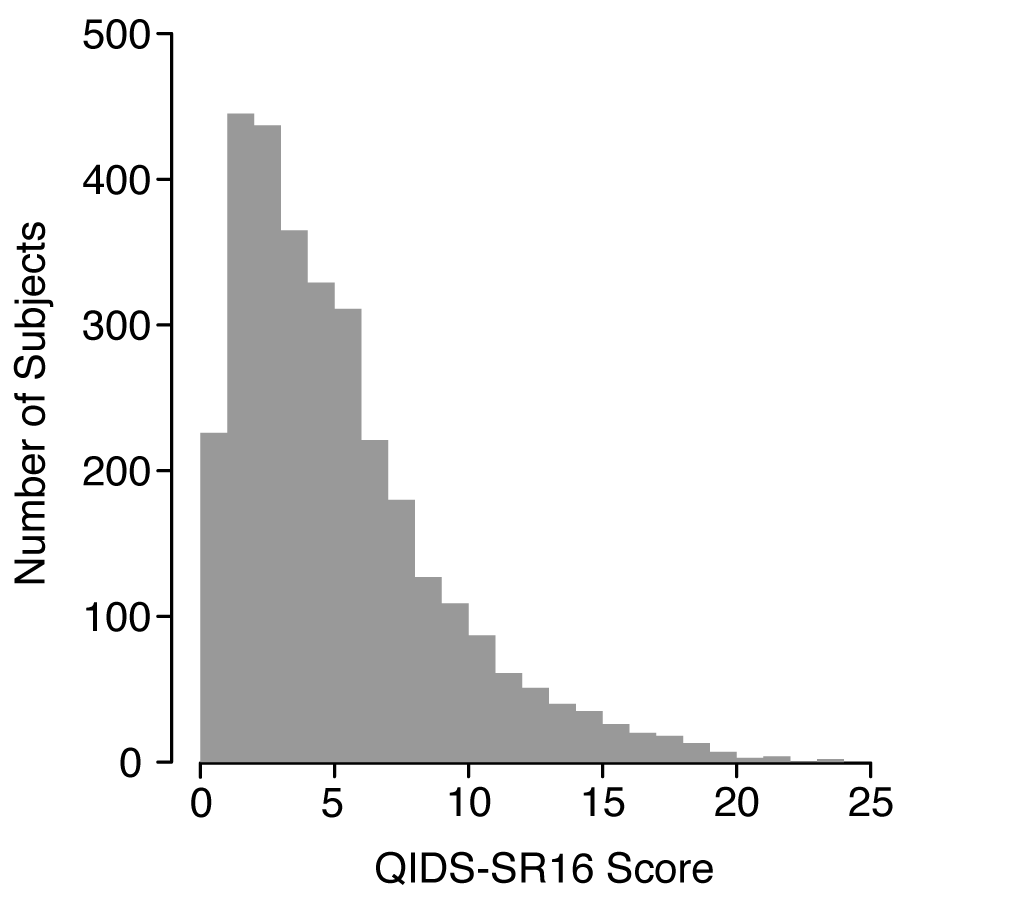

Supplement: S1 Fig — (TIF) [file pone.0184382.s001.tif]
